# Solitary fibrous tumor of the soft palate: A report of two cases

**DOI:** 10.3892/ol.2014.1977

**Published:** 2014-03-14

**Authors:** XUE-MING LI, JIAN-QUN YU, GUO-HUI XU

**Affiliations:** 1 Department of Radiology, Sichuan Cancer Hospital and Institute, Sichuan University, Chengdu, Sichuan 610041, P.R. China; 2 Department of Radiology, West China Hospital, Sichuan University, Chengdu, Sichuan 610041, P.R. China

**Keywords:** solitary fibrous tumor, soft palate, computed tomography

## Abstract

Solitary fibrous tumors (SFTs) are a unique group of mesenchymal neoplasms of fibroblastic or myofibroblastic origin and are extremely rare in the oral cavity. The present study reported two additional cases of SFTs in the soft palate, along with the computed tomography characteristics, which demonstrated well-circumscribed soft tissue lesions with marked and homogenous enhancement. Following wide resection, one of the cases showed certain malignant pathological characteristics, including infiltration of mucinous gland, hypercellularity, nuclear atypia and weak positive staining for S-100. In our experience, SFTs should be considered as a differential diagnosis when a well-circumscribed and solid mass in the soft palate is identified.

## Introduction

Solitary fibrous tumors (SFTs) are a unique group of mesenchymal neoplasms, originally reported in the pleura or other serosal sites (such as peritoneum and pericardium). SFTs in the palate are distinctly uncommon. To the best of our knowledge, only four cases of SFTs located in the palate have been reported in the literature to date, including three in the hard palate and one in the soft palate (1–4). All of these cases were excised with no recurrence and none exhibited specific radiological characteristics. To further investigate the occurrence of this uncommon tumor in this location, the present study reports two additional cases of SFTs in the soft palate and their computed tomography (CT) findings. Written informed consent was obtained from both patients.

## Case reports

### Case 1

A 66-year-old woman was referred to the Sichuan Cancer Hospital and Institute (Chengdu, China) presenting with a soft palatal mass that was discovered following oral examination for sudden pharyngeal discomfort 20 days previously. Physical examination was unremarkable, with the exception of the mass, and the laboratory results showed no obvious abnormalities.

**Figure 1:**
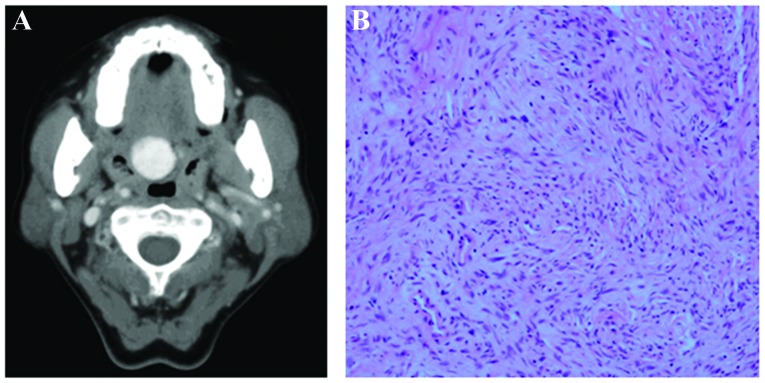
Imaging and pathological findings of the solitary fibrous tumor in Case 1. (A) A well-circumscribed mass with homogenous enhancement measuring ~2 cm in diameter was detected in the soft palate on an enhanced computed tomography scan. (B) Hematoxylin and eosin-stained section of tumor showing storiform or fascicular pattern (original magnification, ×200).

A plain CT scan confirmed a well-circumscribed, round soft tissue mass measuring ~2 cm in diameter at the soft palate. The tumor showed marked and homogeneous enhancement following intravenous injection of contrast material, no evidence of bone invasion and enlarged cervical nodes were found (Fig. 1A). A benign lesion of pleomorphic adenoma was suspected and the tumor was excised with considerable local resection. The surgical specimen was oval shaped and encapsulated by relatively dense fibrous tissue; microscopic examination revealed a storiform or fascicular pattern (Fig. 1B) that was consistent with the diagnosis of an SFT. The patient was well and free of disease 20 months after surgery.

### Case 2

**Figure 2:**
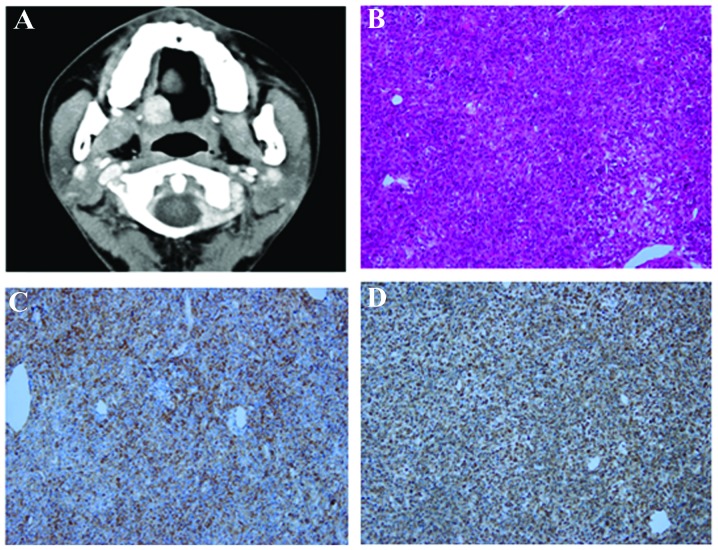
Imaging and pathological findings of the solitary fibrous tumor in Case 2. (A) A well-circumscribed nodule with homogenous enhancement measuring ~1.5 cm in diameter was detected in the right side of soft palate on enhanced computed tomography scan. (B) Hematoxylin and eosin-stained section of the tumor showing hypercellular haphazard spindle-cell proliferation with a small amount of nuclear atypia and abundant capillary vessels (original magnification, ×200). Photomicrograph showing diffuse and intense reactivity with (C) cluster of differentiation 34 and (D) vimentin (original magnification, ×200).

A 27-year-old woman presented with an asymptomatic submucosal mass involving the right soft palate. A soft tissue nodule measuring ~1.5 cm in diameter was detected in the right side of the soft palate on a plain CT scan. The lesion showed marked and homogeneous enhancement following intravenous injection of contrast material, and no evidence of bone invasion and enlarged cervical nodes was found (Fig. 2A). The patient underwent wide local excision. Microscopic examination (Fig. 2B) revealed hypercellular, haphazard spindle cell proliferation with a small amount of nuclear atypia, abundant capillary vessels and invasion of mucinous gland. Immunohistochemically, the specimen was diffuse and showed intense positive staining for cluster of differentiation (CD)34 (Fig. 2C) and vimentin (Fig. 2D), partial positive staining for B-cell lymphoma 2 (Bcl-2), CD99, epithelial membrane antigen (EMA) and smooth muscle actin (SMA), weak positive staining for S-100, but negative for desmin. The patient was diagnosed with an SFT with malignant characteristics. The patient was free of disease 16 months after surgery.

## Discussion

SFTs were first described by Klemperer and Rabin in 1931 as a localized fibrous mesothelioma (5). SFTs are currently considered as pathologically diverse, ubiquitous mesenchymal neoplasms of fibroblastic or myofibroblastic origin and may originate from any site of the body (6,7). Previously, the majority of SFTs were incorrectly characterized as hemangiopericytomas; however, the new World Health Organization (WHO) classification of soft tissue tumors now classifies most hemangiopericytomas as SFTs (6,7). Oral cavity involvement of this disease is rare, with ~80 cases reported in the literature to date (8); generally, SFTs are reported to be slow-growing, painless, well-circumscribed and exhibit mobile submucosal growth of variable size and duration.

Grossly, SFTs are usually well-demarcated and partially encapsulated neoplasms (7). Microscopically, SFTs show a wide range of morphological characteristics from predominantly fibrous lesions containing alternating fibrous areas and hyalinized thick-walled vessels to more cellular and less fibrous neoplasms with a ‘patternless pattern’ (a monotonous appearance) and thin-walled branching vessels (7). Immunohistochemically, SFTs usually manifest vimentin, CD34 and CD99, with variable Bcl-2, EMA and SMA positivity; but are usually negative for CD68, pan-cytokeratins, desmin and S-100 protein immunoreactivity (4,7). General histological characteristics that may enable identification of a malignant lesion include infiltrative margins, hypercellularity, nuclear atypia, necrosis and numerous mitoses (≥4 mitoses per 10 high power fields) (7). In addition, malignant SFTs tend to lose CD34 immunoreactivity and overexpress p53 and S-100 (9). One of our patients showed certain malignant pathological characteristics, including infiltration of mucinous gland, hypercellularity, nuclear atypia, and was weakly positive for S-100. To the best of our knowledge, this is the first case of palatal SFT with malignant characteristics. The behavior of extra-pleural SFTs is unpredictable. Local recurrences and distant metastases may be expected in malignant SFT; however, certain cases of ‘histologically benign’ SFTs do recur or metastasize (8,10). Therefore, long-term follow-up of all the patients with SFT is highly recommended, regardless of the anatomical location.

The majority of cases of SFT are discovered through an incidental radiological finding or as a result of symptoms associated with a mass effect (11). Imaging, such as CT, often provides the first clue in identifying these tumors, depiction of local extent and invasion of adjacent structures, which is useful in guiding surgery. CT imaging demonstrates SFTs as well-circumscribed, hypervascular masses with varying degrees of enhancement, necrosis or cystic change, and occasional internal calcification may be presented (12). In the present study, the two patients demonstrated well-circumscribed, homogenous lesions with marked and homogenous enhancement, which was compatible with a benign tumor. Palatal masses represent a number of diseases, including benign lesions, such as salivary gland tumors, hemangioma and inflammatory diseases, and malignant tumors originating from the oral mucosa. In general, malignant tumors have irregular contours and may be associated with lymphadenopathy; whereas benign lesions tend to have a regular shape and lymphadenopathy is absent. However, radiological differentiation of SFTs from other salivary gland tumors, such as pleomorphic adenoma may be impossible. Thus, currently, SFTs cannot be diagnosed solely from imaging findings and a combination of histological and immunohistochemical examinations are required.

In summary, the present study reported two additional cases of SFTs involving the soft palate and described their CT findings. One case showed certain malignant pathological characteristics. Thus, we suggest when a well-circumscribed and solid mass in the soft palate is found, SFT should be included in the differential diagnosis.
